# 540. A Dose-Comparison Study of Methylprednisolone on Mortality in COVID-19 Patients Require Oxygen in A Tertiary Hospital in Thailand

**DOI:** 10.1093/ofid/ofad500.609

**Published:** 2023-11-27

**Authors:** Pattraporn Piyapan, Viranpat Masamhisak

**Affiliations:** Bhumibol Adulyadej Hospital, Bangkok, Krung Thep, Thailand; Bhumibol Adulyadej Hospital, Bangkok, Krung Thep, Thailand

## Abstract

**Background:**

Dexamethasone 6 mg IV for 10 days or an equivalent dose of another corticosteroid like methylprednisolone (MP) is recommended for severe COVID-19 patients. A higher dose has been reported as beneficial for severe patients. We aimed to compare the effect of < 3 and ≥ 3 mg/kg/day (MKD) of MP in patients who require conventional oxygen therapy or high-flow nasal cannula

**Methods:**

A retrospective study was conducted between June and October 2022 in Bhumibol Adulyadej Hospital by reviewing the medical record of COVID-19 patients requiring conventional oxygen therapy or high-flow nasal cannula. The patients were divided into two groups by the dosing of MP to ≥ 3 MKD and < 3 MKD to compare baseline characteristics, treatment, and clinical course. Multivariate logistic regression was used to identify factors associated with mortality.

**Results:**

There are 197 patients included in the study: 100 from ≥ 3 MKD group and 97 from < 3 MKD group. The mean daily dose of MP was 4.16 (± 0.93) and 2.25 (± 0.55) MKD. There were no statistical differences between the baseline characteristic of the two groups except enoxaparin prophylaxis were more used and LDH were higher in ≥ 3 MKD group. Mortality at 28 days was 26% in ≥ 3 MKD group vs 8.2% in < 3 MKD group (adjusted odd ratio, 3.52 [95% CI, 1.44-8.62]; *P* = 0.006). Age > 65 is also an independent factor associated with mortality (*P* = < 0.001). Bacteremia occurred in 10% VS 2% of ≥ 3 and < 3 MKD groups, respectively (odd ratio, 0.19 [95% CI, 0.04-0.89]; *P* = 0.02).

**Conclusion:**

During the fourth wave of the COVID-19 pandemic with the Delta variant in Thailand, we found patients requiring conventional oxygen therapy or high-flow nasal cannula who received a higher dose of MP (≥ 3 MKD) were associated with significant 28-day mortality.

**Disclosures:**

**All Authors**: No reported disclosures

